# Supplementary material for: Baculovirus Display of Varicella–Zoster Virus Glycoprotein E Induces Robust Humoral and Cellular Immune Responses in Mice
Source: Viruses. 2022 Aug 16;14(8):1785. doi: 10.3390/v14081785 (PMC9416595; doi:10.3390/v14081785)
Supplement: Supplementary file 1 [file viruses-14-01785-s001.zip › viruses-1876853-supplementary.pdf]

Supplementary Materials

Baculovirus Display of Varicella-Zoster Virus Glycoprotein E induces Robust Humoral and Cellular Immune Responses in Mice

Wenhui Xue [1,2,†], Tingting Li [1,2,†], Sibo Zhang [1,2,†], Yingbin Wang [1,2,†], Minqing Hong [1,2], Lingyan Cui [1,2], Hong Wang [1,2], Yuyun Zhang [1,2], Tingting Chen [1,2], Rui Zhu [1,2], Zhenqin Chen [1,2], Lizhi Zhou [1,2], Rongwei Zhang [1,2], Tong Cheng [1,2], Qingbing Zheng [1,2], Jun Zhang [1,2], Ying Gu [1,2,*], Ningshao Xia [1,2,*] and Shaowei Li [1,2,*]

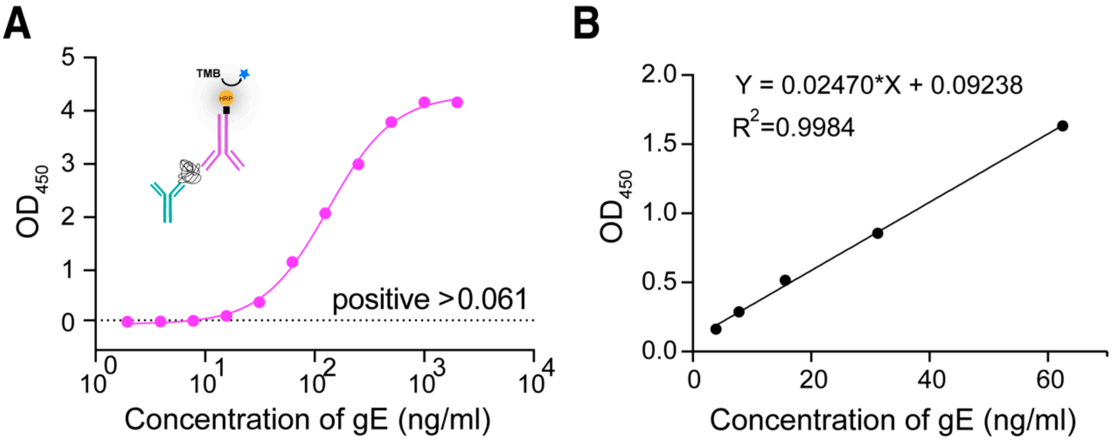

Figure S1. The quantitative estimation of gE proteins was executed by sandwich ELISA. (**A**) Sandwich ELISA using gE-specific antibody 4A2 as the capture antibody and 6H6 (HRP-labeled) as the detection antibody to quantitatively determine the level of gE protein expression. (**B**) Membrane-anchored gE concentration as determined using the established linear curve (R^2 = 0.9984).

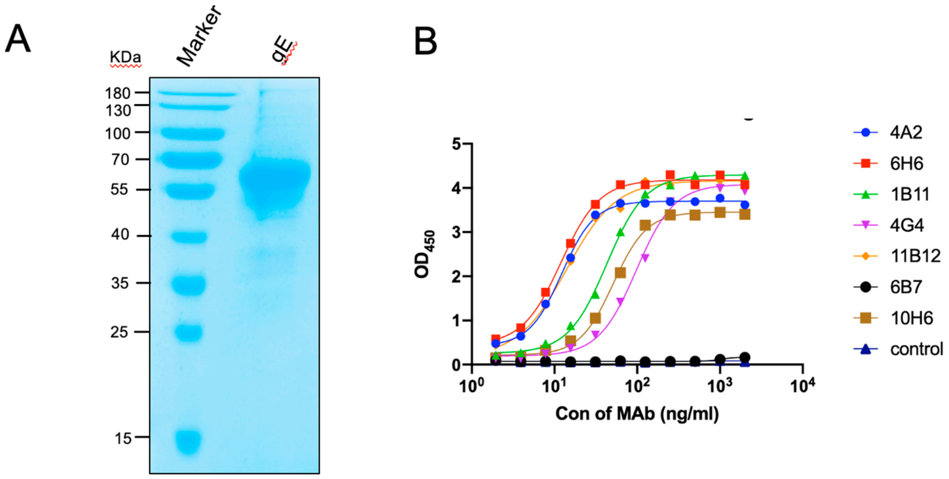

Figure S2. Preparation and Characterization of gE proteins. (**A**) SDS-PAGE analysis of recombinant gE protein produced by insect cells. (**B**) Binding reactivities of recombinant gE protein to VZV-specific antibodies.
